# High SALM3 Expression in Tumor Cells and Fibroblasts Is Correlated with Poor Prognosis in Gastric Cancer Patients

**DOI:** 10.1155/2019/8282414

**Published:** 2019-04-04

**Authors:** Ying Liu, Xiaoli Chen, Xi Chen, Xiaobing Yang, Qingjie Song, Han Wu

**Affiliations:** ^1^Department of Pathology, The Affiliated Hospital of Nantong University, Jiangsu Province 226001, China; ^2^Department of Pathology, The First People's Hospital of Nantong, Jiangsu Province 226001, China; ^3^Department of General Surgery, The Affiliated Hospital of Nantong University, Jiangsu Province 226001, China

## Abstract

**Objective:**

The synaptic adhesion-like molecule (SALM) family is largely restricted to neural tissues and is involved in the regulation of neurite outgrowth and synapse formation. However, the expression of SALM3 in gastric cancer (GC) and its clinical significance remain unclear. The aim of the present study was to investigate the prognostic value of SALM3 in patients with GC.

**Patients and Methods:**

Expression of SALM3 was validated by tissue microarrays from 730 GC patients and statistically assessed for correlations with the clinical parameters and the prognosis of the patients. The transcriptional and survival data of SALM3 in GC patients were also mined through the Oncomine and Kaplan-Meier Plotter databases.

**Results:**

SALM3 is overexpressed in the tumor cells and fibroblasts of clinical GC tissues, and a high level of SALM3 was significantly associated with tumor invasive characteristics. Cox proportional hazards univariate and multivariate regression analyses revealed SALM3 expression in tumor cells or stroma as an independent prognostic factor in the overall survival rate of GC patients. Furthermore, the survival of GC patients with high SALM3 expression in both tumor cells and fibroblasts was significantly poorer than that of the other groups. Oncomine and Kaplan-Meier Plotter analyses further confirmed high levels of SALM3 expression in GC, and high levels of SALM3 expression were associated with shorter survival in patients.

**Conclusion:**

SALM3 may be a prognostic factor for GC and may potentially be a high-priority therapeutic target.

## 1. Introduction

Gastric cancer (GC) is the second leading cause of cancer-associated mortality worldwide, and it is the most common gastrointestinal malignancy in Eastern Europe and East Asia, especially in China [1–3]. Although considerable advances have been achieved in early diagnosis, surgical techniques, and medical treatment, more than half of patients at the advanced stage of the disease die of cancer recurrence and metastasis, even after receiving radical gastrectomy [4]. Postoperative recurrence and metastasis are the biggest obstacles to the treatment of GC [5]. In view of the high frequency of new cases and the adverse outcomes in GC, there has been an exploration for biologic markers that are associated with the development and prognosis of this disease. Nevertheless, to date, no such markers have been found as ideal clinical predictive factors for the diagnosis, therapy, or prognosis of GC. Therefore, it is essential to identify novel prognostic and predictive markers, which will aid in novel effective therapies for GC.

The synaptic adhesion-like molecule (SALM) family of adhesion molecules, also known as Leucine-rich repeat and fibronectin type III domain-containing (LRFN), belongs to the superfamily of leucine-rich repeat-containing adhesion molecules [6, 7]. Previous reports showed that the protein expression of the SALM family, which includes five known members, is largely restricted to neural tissue, and these proteins play vital roles in neuritis growth and branching and synapse formation and maturation [8, 9]. However, recently, some research has demonstrated that SALM family members are also expressed and function in nonneural tissues. Some studies showed that SALM1/LRFN2 was involved in erythropoiesis [10] and that SALM2/LRFN1 participates in pancreatic cancer cell survival [11]. Furthermore, Konakahara et al. [12] demonstrated that SALM3/LRFN4 expression in monocytic cell line THP-1 was upregulated with macrophage differentiation. Additionally, SALM3/LRFN4 signaling plays a vital role in monocyte/macrophage migration [13]. In addition, they also showed that SALM3/LRFN4 was expressed in a variety of human leukemia and cancer cell lines, such as Jurkat, MKN45, SW480, and PANC-1 [12]. Up to now, the expression pattern of SALM3 and its clinical significance in gastric cancer remains poorly understood.

Until recent years, the principal focus of cancer research has mostly been on the malignant cells themselves. In fact, the growth of a tumor is not determined only by cancer cells, because interactions between malignant cells and stromal compartments have a major impact on cancer growth and progression [14]. Advances in understanding the contribution of fibroblasts to cancer progression will enhance our awareness and knowledge about this reciprocal signaling, which supports and promotes the growth, dedifferentiation, invasion, and survival of tumors [15]. More and more studies have specifically showed the expression of tumor markers in fibroblasts and found them to be closely involved in cancer progression and patients' prognoses [16]. Kessenbrock et al. [17] speculated on the multiple functions of matrix metalloproteinases (MMPs) in the tumoral stroma and categorized these proteases according to roles in tissue angiogenesis, invasion, and intravasation, as well as in the preparation of the metastatic niche. A better understanding of the cross talk between the cancer cells and the fibroblasts will enhance our knowledge about the growth-promoting signaling pathways and finally lead to new therapeutic interventions targeting the tumor stroma [15].

In the present study, we evaluated the relationship between SALM3 expression in GC cells and tumoral stroma in tissue microarrays (TMAs) by immunohistochemistry (IHC) and clinicopathologic characteristics including prognostic significance. In addition, we explored the expression of SALM3 in gastric cancer *vs*. normal tissues based on the Oncomine databases, as well as its corresponding prognostic value in the Kaplan-Meier Plotter databases.

## 2. Materials and Methods

### 2.1. Patients and Specimens

From January 2004 to November 2009, 730 GC and 20 matched adjacent nontumor tissues were taken from radical surgical procedures; also, 27 chronic gastritis, 26 intestinal metaplasia, 32 low-grade intraepithelial neoplasia, and 25 high-grade intraepithelial neoplasia tissues were acquired through gastric endoscopic biopsies and were randomly obtained from the clinical biobank of the Affiliated Hospital of Nantong University. Of the samples, none of the 730 GC patients had received any type of treatment before surgery. All GC patients were observed until March 2017, with a median observation time of 42.5 months. Follow-up procedures were described in our previous studies [18]. At the last follow-up, 314 (43.01%) patients had died from either recurrence of the disease (*n* = 274) or surgery-related complications without recurrence (*n* = 140). Among the remaining 416 patients, the mean duration of follow-up was 72.5 months (range: 17.4-130.4 months, standard deviation: ±11.2). Overall survival (OS) was defined as the interval between the dates of surgery and death. Progression-free survival (PFS) was defined as the interval between the dates of surgery and recurrence; if recurrence was not diagnosed, patients were censored on the date of death or the last follow-up. The study protocol conformed to the ethical guidelines of the 1975 Declaration of Helsinki and was approved by the Human Research Ethics Committee of our hospital. Written informed consent was obtained from all study participants.

### 2.2. TMA Construction and Immunohistochemistry Analysis

TMAs were constructed as our previous reports [19]. Briefly, two cores from representative blocks of the formalin-fixed and paraffin-embedded tissues were used to construct TMA slides using the manual Quick Ray Tissue Microarrayer System (UT06, Unitma Co. Ltd., South Korea), which we have in the Department of Clinical Pathology of our hospital.

IHC was carried out using a rabbit polyclonal anti-human SALM3 antibody (1 : 80, MAB5445, R&D Systems, Minneapolis, MN, USA) with a EnVision+™ peroxidase kit (Dako, Carpinteria, CA, USA). Secondly, samples were incubated with 3,3′-diaminobenzidine (Dako, Carpinteria, CA, USA). Negative controls were performed identically but without the primary antibodies. SALM3 staining was semiquantitatively assessed using the H-score method [20] depending on the scores of staining intensity (0 as no staining, 1+ as weak staining, 2+ as moderate staining, and 3+ as intense staining) and the scores of percentages of positive tumor cells (0 as 0-20%, 1 as 21-50%, 2 as 51-70%, and 3 as 71-100%). The final IHC scores were defined as the product of staining intensity and percentages which was calculated as ranging from 0 to 300. All scores of cases were reviewed and calculated by two independent pathologists without any knowledge of the clinical characteristics.

### 2.3. Oncomine Analysis

To determine the SALM3 expression pattern in GC, we used the datasets in the Oncomine Cancer Microarray Database (https://www.oncomine.org) [21]. In order to analyze the messenger ribonucleic acid (mRNA) levels of SALM3 in GC, the mRNA expressions of SALM3 in clinical cancer specimens were compared with those in normal controls, with a Student *t*-test to generate a *p* value. The fold change and the cutoff of the *p* value were defined as 2 and 0.01, respectively.

### 2.4. The Kaplan-Meier Plotter

To analyze the prognostic value of the mRNA expression of SALM3, we used the Kaplan-Meier Plotter (http://www.kmplot.com) [22], which includes the gene expression data and survival information of 1,065 clinical gastric cancer patients [23]. Depending on the median expression of SALM3, we analyzed the PFS and OS of patients with GC who were divided into two cohorts with low and high expression, by the hazard ratio (HR) with log-rank *p* value and 95% confidence intervals (CI).

### 2.5. Statistical Analysis

All analyses were conducted with the SPSS 20.0 software (IBM Corporation, Armonk, NY, USA). The X-Tile software (Rimm Lab, Yale University School of Medicine, New Haven, USA) was used to analyze the cutoff values for low or high SALM3 levels. Pearson's chi-square test was used to determine the correlation between the expression of SALM3 and clinicopathological parameters. The Kaplan-Meier method and the log-rank survival analysis were used to generate the survival curves. Univariate and multivariate analyses were performed with a Cox proportional hazards model to identify the prognostic factors. *p* values < 0.05 were considered statistically significant.

## 3. Results

### 3.1. SALM3 Protein Expression in GC by IHC

**Figure 1:**
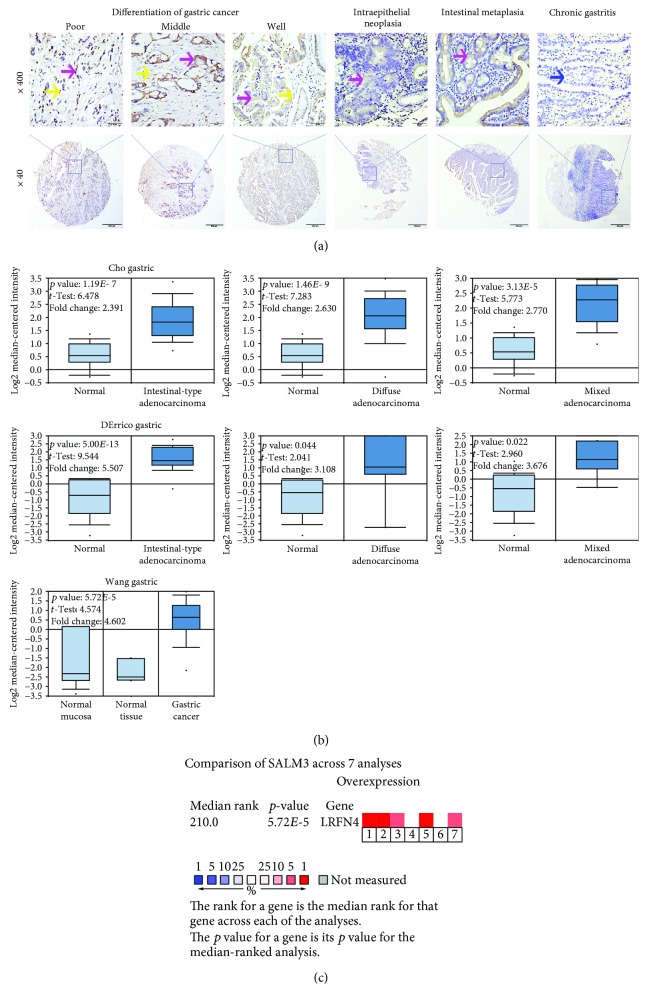
High expression of SALM3 in gastric cancer cells and fibroblasts. (a) Representative images of SALM3 expression in benign and malignant gastric tissue samples: positive tumor cytoplasm (purple arrow), fibroblasts (yellow arrow), and negative staining (blue arrow) of immunohistochemical staining of SALM3. Rows 1 and 2 are SALM3 staining at a magnification of 400x (bar = 50 *μ*m) and 40x (bar = 500 *μ*m), respectively. (b) Box plots from gene expression data in Oncomine comparing the expression of SALM3 in normal and GC tissues. The *p* value was set up at 0.01, and fold change was defined as 2. (c) A meta-analysis of SALM3 gene expression from 3 Oncomine databases, where colored squares indicate the median rank for SALM3 (vs. normal tissue) across 7 analyses: Cho's gastric (1-3), DErrico's gastric (4-6), and Wang's gastric (7). The *p* value is given for the median-rank analysis.

To investigate the SALM3 expression in clinical GC tissues, we performed IHC analysis with TMAs, which contained matched nontumor tissues and complete clinical outcome information. Positive staining of SALM3 was primarily localized in tumor cell cytoplasm and membranes and in fibroblasts. Representative IHC SALM3 staining patterns are shown in Figure 1(a). Furthermore, we used the X-Tile software to measure the cutoff values for high or low SALM3 expression. Here, 60 was determined as the cutoff point for SALM3 in tumor and stromal cells; scores from 0 to 60 were considered as low expression and scores from 61 to 300 were deemed as high expression. In cancer cells, high SALM3 protein levels were detected in 340 (46.6%) of 730 GC tissues and showed statistical significance (*χ*^2^ = 62.87, *p* < 0.001). In addition, a significantly high SALM3 expression in stromal cells was identified in 261 (35.8%) of 730 GC tissues (*χ*^2^ = 59.39, *p* < 0.001) (Table 1).

### 3.2. Database Analysis Reveals That SALM3 Is Upregulated in GC

To determine the clinical significance of SALM3 in patients with GC, we performed data mining and analyzed SALM3 mRNA levels from the publicly available Oncomine database. The finding for SALM3 mRNA expressions based on 6 databases identified 3 with a significant *p* value (*p* < 0.001), and this gene ranks in the top 10% among all differentially expressed genes. We collected the results from Cho's [24], DErrico's [25], and Wang's [26] studies and analyzed SALM3 mRNA expression in GC. SALM3 expression was found higher in gastric normal mucosa than in cancer, even when stratified into intestinal-, diffuse-, and mixed-type carcinomas by Lauren's classification (*p* < 0.05, Figure 1(b)).

The median rank of SALM3 in upregulated genes of GC was 210.0 based on a meta-analysis across the three above datasets, including 7 analyses using Oncomine algorithms [27] (90 + 69 + 27 + 502 samples, *p* = 5.72*E* − 5, Figure 1(c)).

### 3.3. Expression Level of SALM3 in Tumor Cells and Fibroblasts and GC Patients' Survival

**Figure 2:**
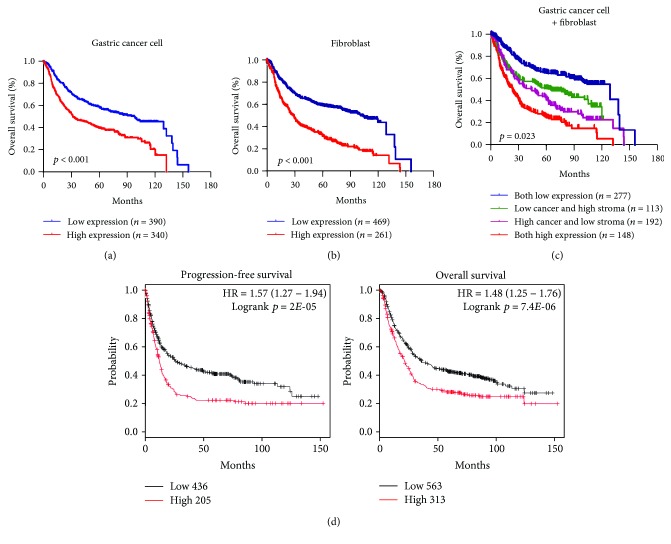
The prognostic values of SALM3 in gastric cancer. High levels of SALM3 in tumor cells (a), stroma (b), and four groups of tumor cells and stroma (c) were associated with shorter overall survival in GC patients of the Nantong cohort. Four groups were classified according to the SALM3 expression in tumor cells and fibroblasts. (d) Based on the data from Kaplan-Meier Plotter, high SALM3 mRNA levels were positively related to both progression-free survival and overall survival of patients with GC.

Among the 730 GC patients, the Kaplan-Meier survival analysis indicated that the high expression of SALM3 in tumor cells (*p* < 0.001) or stroma (*p* < 0.001) was significantly associated with poor OS (Figures 2(a) and 2(b)). The multivariate analysis demonstrated that in addition to conventional clinicopathological parameters, such as nodal status and metastasis, SALM3 in tumor cells (*p* < 0.001) and fibroblasts (*p* < 0.001) was an independent unfavorable factor for OS (Table 2). To evaluate the combined effect of SALM3 on the prognosis of GC, we classified patients into four subgroups according to the SALM3 expression in tumor cells and fibroblasts: group I had low expression of the two distributions, group II had low tumor cell and high fibroblast expression, group III had high tumor cell and low fibroblast expression, and group IV had a high expression of both distributions. We found that the OS of group IV was significantly lower than that of the other groups (*p* = 0.023, Figure 2(c)).

We also used the Kaplan-Meier Plotter to analyze the prognostic significance of SALM3 mRNA. Based on the data from the Kaplan-Meier Plotter, SALM3 mRNA expression was positively correlated to both OS and PFS rates of the patients with GC (Figure 2(d), *p* < 0.05).

### 3.4. Upregulation of SALM3 Is Correlated with Advanced Clinicopathological Features of GC

We analyzed the correlation between SALM3 expression and the clinicopathological characteristics of GC. Strong associations were observed between SALM3 expression in tumor cells and tumor classification (*p* < 0.001), lymph node metastasis (*p* = 0.019), tumor metastasis (*p* = 0.004), and TNM stage (*p* < 0.001) (Table 3). High SALM3 expression in fibroblasts was significantly associated with tumor classification (*p* < 0.001), lymph node metastasis (*p* < 0.001), and TMN stage (*p* < 0.001) (Table 3). These results significantly indicated a correlation between the expression of SALM3 and an unfavorable prognosis of GC.

## 4. Discussion

In the present study, we revealed that SALM3 was highly expressed in GC and fibroblasts and was significantly associated with clinical parameters and reduced survival time of patients with GC. Multivariate analysis showed that SALM3 expression in GC cells and fibroblasts might be an independent prognostic factor of survival in patients with GC.

Some research has previously demonstrated that the expression and function of the members of the SALM family are mostly restricted to neural tissues [28]. Nevertheless, a recent study reported that SALM3, also known as LRFN4, is expressed in some cancer cell lines, such as Panc-1, JURKAT-1, and MKN7 [12]. In the present study, we found that protein expression of SALM3 was primarily localized in the gastric cancer cell cytoplasm. We also found that SALM3 protein expression in cancer samples was higher than that in paracancer tissues and benign gastric disease tissues. Moreover, high SALM3 expression in GC was associated with certain clinicopathological characteristics, such as primary cancer, distant metastasis, and TNM stage. Our results showed that SALM3 plays a protumorigenic role in gastric cancer. Consistent with this conclusion, we found that the upregulation of SALM3 is generally correlated with an adverse prognosis of gastric cancer patients. Oncomine data expression analysis also showed that SALM3 is upregulated in gastric adenocarcinoma tumor, which provides another layer of evidence that SALM3 might positively take part in the regulation and development of GC.

The cross talk between the cancer cells and tumoral stroma is significantly associated with the progression and metastasis of tumor [29]. During recent years, many studies have specifically identified and demonstrated some tumor markers expressed in the tumor stroma that are closely correlated with tumor progression and patients' adverse prognoses. In the present study, we found that high SALM3 expression in the fibroblasts of gastric cancer tissues was related with regional lymph node metastasis and advanced TNM stage, and it independently predicted unfavorable OS for cancer patients. Furthermore, in terms of SALM3 expression in cancer cells and stroma, GC patients with cancer-cells^high^ and fibroblasts^high^ had worse prognosis than the other groups. Cancer is composed of not only simply autonomous malignant cells but fibroblasts, endothelial cells, immune cells, and specialized mesenchymal cells. The neoplastic cells can recruit these different types of stroma cells to facilitate the growth of the tumor and contribute to distant metastasis [30]. Importantly, tumor-infiltrating immune cells, especially the presence of macrophages at the margins of tumors, have been noted to be significantly related to the stimulation of cancer cell proliferation, tissue invasion, and support of cancer cell seeding and further metastatic dissemination, via inducing and helping sustain tumor angiogenesis [23, 31]. Interestingly, it was reported that SALM3 expression is upregulated in monocytic cells with macrophage differentiation [12]. Additionally, SALM3 signaling plays a vital role in inducing the migration of monocytes/macrophages into the inflammation area [12, 13]. Therefore, we speculate that SALM3 might participate in the procedure of dissemination and recurrence of gastric cancer.

Nevertheless, there are several limitations in our research. We need to apply further larger prospective studies to the general population for confirmation to correct the shortcomings of a retrospective observational study. Additionally, as a semiquantitative IHC data study, it needs additional methods to evaluate and confirm SALM3 expression in tumor cells and stroma. Furthermore, we should investigate the mechanisms of SALM3 in tumorigenesis by *in vitro* studies in the next step.

## 5. Conclusions

We found that SALM3 is upregulated in gastric cancer tissues and SALM3 expression is negatively correlated with patients' survival. In a word, it is suggested that SALM3 can serve as a potential marker for predicting clinical prognosis and a therapeutic target for gastric cancer patients.

## Figures and Tables

**Table 1:** SALM3 expression in gastric benign and malignant tissues.

Characteristic	*n*	SALM3 in tumor cells				SALM3 in fibroblasts			
		SALM3- (%)	SALM3+ (%)	Pearson *χ*^2^	*p*	SALM3- (%)	SALM3+ (%)	Pearson *χ*^2^	*p*
Total	860	511 (59.4)	349 (40.6)	62.87	<0.001	596 (69.3)	264 (30.7)	59.39	<0.001
Chronic gastritis	27	26 (96.3)	1 (3.7)			27 (100.0)	0 (0.0)		
Intestinal metaplasia	26	25 (96.2)	1 (3.8)			26 (100.0)	0 (0.0)		
Low-grade intraepithelial neoplasia	32	29 (90.6)	3 (9.4)			32 (100.0)	0 (0.0)		
High-grade intraepithelial neoplasia	25	22 (88.0)	3 (12.0)			22 (88.0)	3 (12.0)		
Matched tumor neighbor	20	19 (95.0)	1 (5.0)			20 (100.0)	0 (0.0)		
Cancer	730	390 (53.4)	340 (46.6)			469 (64.2)	261 (35.8)		

^∗^ *p* < 0.05; SALM3- represents low expression and SALM3+ represents high expression.

**Table 2:** Univariate and multivariate analysis of prognostic factors for overall survival in gastric cancer patients.

	Univariate analysis			Multivariate analysis		
	HR	*p* > ∣*z*∣	95% CI	HR	*p* > ∣*z*∣	95% CI
Gender						
Male vs. female	1.014	0.903	(0.816, 1.258)			
Age						
≤60 vs. >60	1.167	0.116	(0.963, 1.413)			
Histological type						
Tubular vs. mucinous vs. mixed (tubular and mucinous) vs. signet ring cells vs. others^a^	1.018	0.698	(0.930, 1.114)			
Differentiation						
Well vs. middle vs. poor	1.526	<0.001^∗^	(1.273, 1.829)	1.096	0.366	(0.898, 1.339)
T						
Tis vs. T1+T2 vs. T3+T4	2.723	<0.001^∗^	(2.207, 3.360)	1.317	0.083	(0.964, 1.798)
N						
N0 vs. N1 vs. N2 vs. N3	1.700	<0.001^∗^	(1.568, 1.844)	1.265	0.002^∗^	(1.093, 1.463)
M						
M0 vs. M1	3.503	<0.001^∗^	(2.556, 4.800)	2.176	<0.001^∗^	(1.534, 3.088)
TNM stage						
0+I vs. II vs. III+IV	2.686	<0.001^∗^	(2.331, 3.096)	1.453	0.022^∗^	(1.054, 2.004)
SALM3 expression in tumor cells						
High vs. low or none	1.785	<0.001^∗^	(1.473, 2.165)	1.471	<0.001^∗^	(1.210, 1.789)
SALM3 expression in stroma						
High vs. low or none	2.262	<0.001^∗^	(1.868, 2.740)	1.525	<0.001^∗^	(1.247, 1.865)
Combination of tumor cells and fibroblasts						
I vs. IV	2.137	0.023^∗^	(1.124, 2.537)	1.621	0.036^∗^	(1.312, 2.472)
II vs. IV	1.276	0.616	(0.714, 2.328)			
III vs. IV	1.769	0.548	(1.357, 2.664)			

^∗^ *p* < 0.05; ^a^Others: papillary adenocarcinoma, 5 cases; adenosquamous carcinoma, 6 cases; squamous cell carcinoma, 7 cases; undifferentiated carcinoma, 1 case; small cell malignant tumor, 9 cases; carcinoid, 2 cases; focal cancer, 2 cases. Four groups were classified according to the SALM3 expression in tumor cells and/or stroma: group I has low expression of both, group II has high expression of tumor cells and low expression of stroma, group III has low expression of tumor cells and high expression of stroma, and group IV has high expression of both.

**Table 3:** Association of SALM3 expression with clinicopathological characteristics in gastric cancer patients.

Characteristic	*n*	SALM3 in tumor cells				SALM3 in fibroblasts			
		SALM3- (%)	SALM3+ (%)	Pearson *χ*^2^	*p*	SALM3- (%)	SALM3+ (%)	Pearson *χ*^2^	*p*
Total	730								
Age				0.233	0.629			1.054	0.305
>60	387	210 (54.3)	177 (45.7)			242 (62.5)	145 (37.5)		
≤60	343	180 (52.5)	163 (47.5)			227 (66.2)	116 (33.8)		
Gender				0.236	0.627			0.031	0.860
Male	537	284 (52.9)	253 (47.1)			344 (64.1)	193 (35.9)		
Female	193	106 (54.9)	87 (45.1)			125 (64.8)	68 (35.2)		
Histological type				5.428	0.246			9.273	0.055
Tubular	614	328 (53.4)	286 (46.6)			403 (65.6)	211 (34.4)		
Mucinous	31	21 (67.7)	10 (32.3)			14 (45.2)	17 (54.8)		
Mixed (tubular and mucinous)	16	7 (43.8)	9 (56.3)			7 (43.8)	9 (56.3)		
Signet ring cell	37	21 (56.8)	16 (43.2)			26 (70.3)	11 (29.7)		
Others^a^	32	13 (40.6)	19 (59.4)			19 (59.4)	13 (40.6)		
Differentiation				5.008	0.082			4.685	0.096
Well	38	27 (71.1)	11 (28.9)			28 (73.7)	10 (26.3)		
Middle	193	101 (52.3)	92 (47.7)			133 (68.9)	60 (31.1)		
Poor	499	262 (52.5)	237 (47.5)			308 (61.7)	191 (38.3)		
T				18.699	<0.001^∗^			29.759	<0.001^∗^
Tis	41	30 (73.2)	11 (26.8)			36 (87.8)	5 (12.2)		
T1+T2	220	136 (61.8)	84 (38.2)			164 (74.5)	56 (25.5)		
T3+T4	469	224 (47.8)	245 (52.2)			269 (57.4)	200 (42.6)		
N				9.958	0.019^∗^			73.214	<0.001^∗^
N0	303	178 (58.7)	125 (41.3)			238 (78.5)	65 (21.5)		
N1	135	72 (53.3)	63 (46.7)			97 (71.9)	38 (28.1)		
N2	139	74 (53.2)	65 (46.8)			64 (46.0)	75 (54.0)		
N3	153	66 (43.1)	87 (56.9)			70 (45.8)	83 (54.2)		
M				8.139	0.004^∗^			2.453	0.117
M0	680	373 (54.9)	307 (45.1)			442 (65.0)	238 (35.0)		
M1	50	17 (34.0)	33 (66.0)			27 (54.0)	23 (46.0)		
TNM stage				19.053	<0.001^∗^			72.728	<0.001^∗^
0+I	184	120 (65.2)	64 (34.8)			149 (81.0)	35 (19.0)		
II	250	137 (54.8)	113 (45.2)			183 (73.2)	67 (26.8)		
III+IV	296	133 (44.9)	163 (55.1)			137 (46.3)	159 (53.7)		

^∗^ *p* < 0.05; ^a^Others: papillary adenocarcinoma, 5 cases; adenosquamous carcinoma, 6 cases; squamous cell carcinoma, 7 cases; undifferentiated carcinoma, 1 case; small cell malignant tumor, 9 cases; carcinoid, 2 cases; focal cancer, 2 cases.

## Data Availability

The data of mRNA levels of SALM3 used to support this study are available at the datasets in the Oncomine Cancer Microarray Database (https://www.oncomine.org). The prognostic data of the mRNA expression of SALM3 supporting this study are from Kaplan-Meier Plotter (http://www.kmplot.com). The IHC data used to support the findings of this study are included within the article.
